# *GABARAPL1* Exerts Regulatory Effects on Hypoxia‐Induced Pyroptosis in the Pathogenesis of Myocardial Infarction

**DOI:** 10.1111/jcmm.70469

**Published:** 2025-03-17

**Authors:** Chunying Liu, Chenghui Yan, Dan Liu, Haixu Song, Yaling Han

**Affiliations:** ^1^ State Key Laboratory of Frigid Zone Cardiovascular Diseases (SKLFZCD), cardiovascular Research Institute and Department of Cardiology Shenyang China; ^2^ Beifang Hospital of China Medical University Shenyang China

**Keywords:** autophagy, *GABARAPL1*, myocardial infarction, PIK90, pyroptosis

## Abstract

Myocardial infarction (MI) is a major health threat, with high incidence and poor prognosis. This study aims to discover novel biomarkers and therapeutic targets to reduce myocardial damage and improve patient survival. A comprehensive bioinformatics analysis of MI datasets was conducted to identify pivotal genes related to pyroptosis and autophagy. These genes underwent protein–protein interaction (PPI) analysis, functional enrichment analysis, and immune infiltration analysis. Receiver operating characteristic (ROC) curves and nomograms were used to pinpoint the most diagnostic hub genes. Western blotting and qRT‐PCR were performed to evaluate their expression and mechanisms. Drug prediction and molecular docking identified potential therapeutic agents targeting hub genes, with validation of their effects on hypoxia‐induced pyroptosis both in vivo and in vitro. In conclusion, GABARAPL1 was identified as a hub gene, and PIK90 emerged as a promising therapeutic candidate drug. GABARAPL1 expression was significantly upregulated in heart tissue following MI and in endothelial cells subjected to hypoxic conditions. Silencing GABARAPL1 aggravated hypoxia‐induced pyroptosis in endothelial cells. In vivo, PIK90 improved survival, reduced cardiac dysfunction, and alleviated myocardial fibrosis induced by MI. In vitro, PIK90 inhibited hypoxia‐induced pyroptosis in endothelial cells. Consequently, *GABARAPL1* may represent a promising therapeutic target for the treatment of MI.

## 1 Introduction

Myocardial infarction (MI) remains the foremost cause of elevated mortality rates on a global scale, and its principal complication significantly impairs patient quality of life and prognosis [1]. Despite diligent monitoring and adherence to guideline‐recommended care, high rates of recurrent ischemic events persist following MI [2, 3]. Oxidative stress, pyroptosis, and apoptosis are among the many variables that contribute to MI [4, 5]. Thus, lowering the incidence of MI and enhancing patient outcomes in the future depend heavily on early MI prediction and a thorough understanding of its pathophysiology.

It is widely recognised that endothelial cells are subjected to diverse mechanical, inflammatory, and metabolic conditions. Elevated levels of low‐density lipoprotein (LDL) cholesterol, pro‐inflammatory states, and disturbed flow patterns can induce endothelial dysfunction, which plays a critical role in the pathogenesis of MI [6, 7]. Pyroptosis is a type of programmed cell death mediated by inflammasomes [8]. The primary characteristics of pyroptosis involve the formation of pores in the cell membrane, leading to cell lysis and the release of pyroptotic bodies [9]. The recent reports further imply the significant involvement of pyroptosis in the pathogenesis of MI. Accumulating evidence suggests that inhibiting NLRP3, IL1β, and IL18 activation has been shown to attenuate inflammation and pyroptosis, thereby mitigating cardiac injury [10]. Autophagy is a highly conserved metabolic pathway that involves the removal of damaged organelles through lysosomes and plays a crucial role in maintaining cellular homeostasis [11]. The physiological state of autophagy remains at a basal level. However, under conditions of stress such as hypoxia or DNA damage, autophagy is upregulated and serves a protective role in the body. Nevertheless, excessive autophagy can result in cellular demise [12]. Recent studies have demonstrated that autophagy and pyroptosis play significant roles in a variety of pathophysiological processes [13, 14]. It has been demonstrated that autophagy plays a pivotal regulatory role in pyroptosis, and these two processes synergistically participate in numerous pathophysiological pathways. Increasing evidence has indicated that the induction of autophagy can suppress myocardial ischemia–reperfusion (I/R)‐induced pyroptosis [15, 16]. The overexpression of Beclin1 mediated I/R injury by promoting autophagy and inhibiting Caspase‐4‐mediated pyroptosis [17]. The involvement and underlying mechanisms of autophagy and pyroptosis in MI remain unexplored in the existing literature.

In this study, we employed a series of bioinformatics to identify the important genes associated with pyroptosis and autophagy in MI datasets. Functional enrichment analysis was conducted on these genes, followed by an investigation into their relationship with immune cells. The diagnostic model recognises *GABARAPL1* as the most valuable gene for diagnosis, and extensive research has been conducted in this regard. The accuracy of our predictions regarding MI was validated through both in vivo and in vitro. In mechanism, GABARAPL1 serves a crucial regulatory function in the process of hypoxia‐induced pyroptosis. Furthermore, the most potential target drug PIK90, performed by drug prediction and molecular docking for *GABARAPL1*, significantly alleviated cardiac dysfunction and myocardial fibrosis induced by MI in mice, and it could inhibit hypoxia‐induced pyroptosis in endothelial cells. Herein, the identification of *GABARAPL1* as a potential novel target holds promise for the prognosis and treatment of MI. The detailed illustration of the analytical workflow is presented in Figure S1.

## 2 Materials and Methods

### 2.1 Data Preprocessing

The GSE66360 dataset, including 49 MI samples and 50 Control samples, was obtained from the GEO database (https://www.ncbi.nlm.nih.gov/gds/), which was employed to explore DEGs. The pyroptosis‐related genes were obtained from the MSIGDB database (http://www.gsea‐msigdb.org/gsea/msigdb/) and the autophagy‐related genes were obtained from the HAMdb2 database (http://www.autophagy.lu/index.html).

### 2.2 Screening of DEGs

A log2|Fold Change (FC)| > 0.585 and adj. *p* < 0.05 were regarded as the standard for screening DEGs. We intersected the 1057 DEGs from the GSE66360 dataset with the 410 selected genes from the pyroptosis database (MSIGDB) and the autophagy database (HAMdb2) to obtain a total of 36 DEGs involved in the autophagy–pyroptosis process in MI.

### 2.3 Building Machine Learning Models

The “caret” R packages were employed to conduct machine learning models, containing random forest model (RF), eXtreme Gradient Boosting (XGB) and support vector machine model (SVM), generalised linear model (GLM), and K‐Nearest Neighbor (KNN). All these five machine learning models were performed with default parameters and assessed via 5‐fold cross validation.

### 2.4 PPI Network

We utilised the GeneMANIA platform (http://genemania.org/) to construct the Protein–Protein Interaction (PPI) network. GeneMANIA is an extensively employed online tool for predicting gene functions and constructing gene interaction networks.

### 2.5 Functional Enrichment Analysis of DEGs

We used the “ClusterProfiler” package in R language to conduct Gene Ontology (GO) and Kyoto Encyclopedia of Genes and Genomes (KEGG) pathway enrichment analyses for functional characteristics of genes in the PPI network.

### 2.6 Gene Set Enrichment Analysis

According to the median number of five important genes, the samples were divided into two groups with high and low expression. The differential genes of each key gene were calculated using the “limma” algorithm, and Gene set enrichment analysis (GSEA) was performed using the “ClusterProfiler” package to check the status of pyroptosis, autophagy, and immune‐related pathways.

### 2.7 Immune Infiltration Analysis

“Pheatmap” package was used to draw heat maps to check the expression of chemokine, Immunostimulator, Immunoinhibitor, MHC, and receptor‐related genes in MI and Control samples. The CIBERSORT algorithm was used to calculate the proportion of 22 infiltrating immune cells in each sample. Violin plots were drawn with the “ggplot2” package to check whether there was a difference in immune cell content between MI and Control samples with *p* < 0.05. The correlation of these genes with immune cells was examined using the lollipop chart (spearman correlation analysis).

### 2.8 Construction of ROC Curve and Nomogram

The “pROC” package was performed to visualise the area under receiver operating characteristic (ROC) curves, and the establishment of a nomogram model was concurrently undertaken to assess the diagnostic efficacy in predicting the incidence of MI.

### 2.9 Prediction of Potential Drug and Analysis of Molecular Docking

CMap (https://clue.io) is a gene expression profile database based on gene expression profile interventions to predict molecular‐targeted drugs. The CMap score was negatively correlated with the potential therapeutic effect of the drug. We uploaded 300 DEGs (150 up‐regulated genes and 150 down‐regulated genes) of *GABARAPL1* to the CMap database to screen the 20 drugs most associated with it. Heat maps are used to demonstrate the mechanism of action (MOA) of these drugs and drug targets to explore their potential mechanisms for treating MI. To further study the binding sites of *GABARAPL1* and its corresponding small molecule compounds, we conducted molecular docking. The 3D structures of the proteins were obtained from the Uniprot database (https://www.uniprot.org/). The molecular docking programs AutoDock Tools [18] and AutoDock vina [19] were used for automatic molecular docking simulation, and the lower the binding energy, the stronger the affinity between the drug molecule and the target protein.

### 2.10 Establish Myocardial Infarction Model

C57BL/6J mice (8w) were used to establish the MI model. The left coronary artery of the mice was ligated under the condition of 2% isoflurane, and the Sham group also underwent the same procedure without coronary artery ligation. Echocardiography was used to testify whether the MI model was successful. The Ethics Committee of North Hospital of China Medical University granted approval for all animal experiments.

### 2.11 Echocardiography

Transthoracic echocardiograms were conducted on mice anaesthetised with 2% isoflurane using the Vevo 2100 ultrasound system (VisualSonics Inc., Toronto, ON, Canada) in M‐mode. Prior to the procedure, chest hair was removed from the mice using a depilatory cream. Cardiac function parameters, including ejection fraction (EF) and fractional shortening (FS), were assessed in M‐mode.

### 2.12 Cell Culture and Treatment

Endothelial cells were cultured in Dulbecco's Modified Eagle Medium (DMEM; Gibco) with 10% fetal bovine serum (FBS; Gibco) and maintained in a 37°C incubator with 5% CO_2_. To induce anoxic conditions, 600 μM cobalt chloride (CoCl_2_) was added to the culture medium for 24 h. Alternatively, to mimic nutrient deprivation, nutrient deprivation was simulated by replacing the normal medium with a solution devoid of both sugars and serum, followed by incubation in a hypoxic chamber with 5% CO_2_ and 95% N_2_ for an additional 24 h.

### 2.13 Transfection

Small interfering RNAs (siRNAs) and a scrambled siRNA (siCtrl) were synthesised by Honorgene (Changsha, China). The target sequences of the siRNAs are presented in Table S1. C166 cells were transfected with siRNAs using Lipo2000 (Invitrogen, Waltham, MA, USA, #11668019) and the subsequent experiments were conducted 24 h after transfection.

### 2.14 Drug Treatments

In vivo, PIK90 (MCE, HY‐12030) was dissolved in a mixture of PEG300 and saline. Mice were intraperitoneally injected with PIK90 at doses of 20 mg/kg/day (Low) or 40 mg/kg/day (High) from Day 1–28 post‐MI. In vitro, PIK90 (MCE, HY‐12030) was dissolved in DMSO, and cells were pre‐treated with 10 μM PIK90 for 1 h prior to hypoxic exposure.

### 2.15 Total RNA Extraction and qRT‐PCR

Total RNA was extracted from heart tissue or the treated C166 and HUVEC cells using TRIZOL reagent. Transcription amplification was performed using a cDNA reverse transcription kit (TaKaRa, #RR047A) and the quantitative analysis was performed. The primers (Sangon Biotech, Shanghai, China) used for detecting were below (Table S2).

### 2.16 Western Blotting

RIPA lysate (Thermo Fisher Scientific, #89901) was used to extract proteins from mouse heart tissue or endothelial cells. The protein concentration was detected by the Pierce bicinchoninic acid (BCA) protein test kit (Thermo Fisher Scientific, #23227). The following proteins were used in Western blotting: GABARAPL1 (1:1000; CST, #26632), Caspase1 (1:1000; Abclonal, A0946), LC3B (1:1000; CST, #89332S), IL1β (1:1000; Abclonal, A16288), NLRP3 (1:1000; HUABIO, #ET1610‐93), GSDMD (1:1000; Proteintech, #20770‐1‐AP), GSDMD/GSDMD‐N antibody (1:1000;Abclonal;A20197), IL18 (1:1000; Abclonal, A23076) and GAPDH (1:1000; CST, #2118). Select the right secondary antibody according to the primary antibody. Signal was detected by the enhanced chemiluminescence (ECL) system (Thermo Fisher Scientific, #34580) and quantified by using Image J software [20].

### 2.17 Histological Staining

Mouse heart tissue was fixed overnight in 4% paraformaldehyde, embedded in paraffin, and sectioned into 5 μm thick slices. The paraffin‐embedded sections were stained with hematoxylin and eosin (H&E) for morphological evaluation of heart tissue. Masson trichromatic staining was employed to evaluate myocardial collagen content and quantify infarct size. Wheat germ agglutinin (WGA) staining was utilised to determine the cross‐sectional area of cardiomyocytes.

### 2.18 Statistical Analysis

Statistical analyses were performed using Graphpad Prism 8. Data were presented as mean ± standard deviation (SD). The Student's *t*‐test and one‐way analysis of variance (ANOVA) were used to evaluate significant differences. *p* < 0.05 was considered statistically significant.

## 3 Results

### 3.1 Identification of DEGs and Enrichment Analysis

**FIGURE 1:**
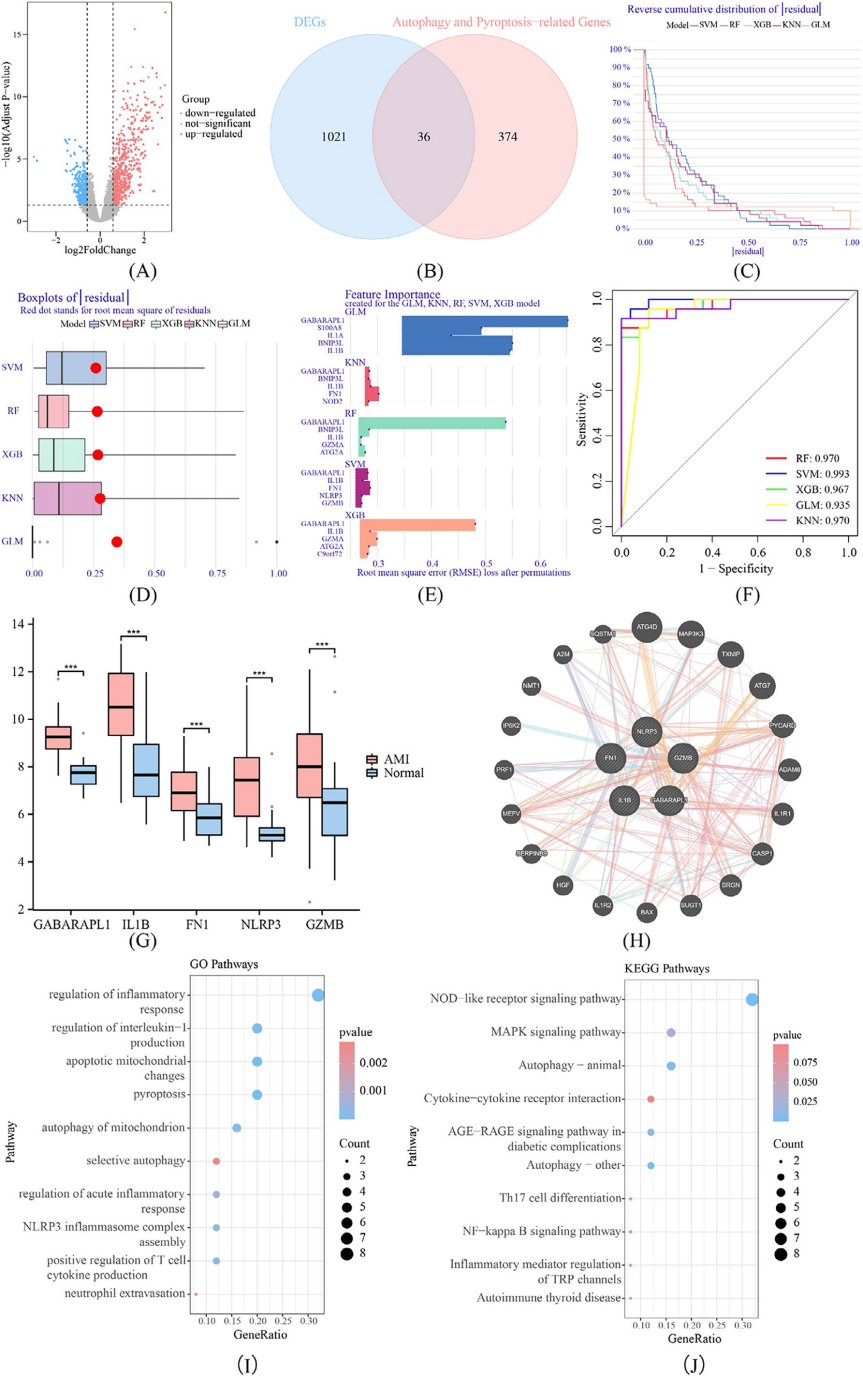
Identification of DEGs. (A) The Volcano plots of DEGs in the GSE66360 dataset. (B) The Venn diagram of DEGs in the GSE66360 and autophagy and pyroptosis‐related genes. (C) Cumulative residual distribution of each machine learning model. (D) Boxplots showing the residuals of each machine learning model. Red dot represented the RMSE values. (E) The important features in KNN, RF, SVM, GLM and XGB machine models. (F) ROC analysis of five machine learning models based on 5‐fold cross‐validation. (G) Box plots showing differential expressions of GABARAPL1, IL1β, FN1, NLRP3 and GZMB in MI and Control samples. ***p < 0.001 vs. Control group. (H) PPI network of the five important genes. (I) GO analysis of the genes in the PPI network. (J) KEGG analysis of the genes in the PPI network.

We first discerned 1057 DEGs within the GSE66360 dataset, with the identification of 647 genes exhibiting upregulation and 410 genes demonstrating downregulation (Figure 1A). Venn diagrams were employed to pinpoint 36 DEGs linked to autophagy and pyroptosis (Figure 1B). To further enhance the accuracy of DEGs, we constructed five machine learning models. The residual distribution of each model was plotted, revealing that the SVM learning model exhibited the lowest residual (Figure 1C,D) and the top five important feature variables of each model were sorted based on root mean square error (RMSE) values (Figure 1E). Additionally, we evaluated the discriminatory performance of these machine learning algorithms by calculating ROC curves. The area under the ROC curve for SVM was highest with AUC = 0.993, followed by RF with AUC = 0.97; XGB with AUC = 0.967; GLM with AUC = 0.935; KNN with AUC = 0.97 (Figure 1F). Based on these findings, it can be conclusively stated that the SVM model excels in distinguishing patients from different clusters. Thus, GABARAPL1, IL1β, FN1, NLRP3, and GZMB were identified as important genes in the development of MI. And the five genes exhibited upregulated expression in MI patients compared with Control samples (Figure 1G). Subsequently, we constructed a PPI network (Figure 1H). To further explore their functional roles, we performed GO and KEGG pathway enrichment analysis. The results from GO analysis demonstrated that these genes were primarily enriched in mitochondrial autophagy, pyroptosis, and inflammatory response pathways (Figure 1I). Additionally, KEGG analysis indicated that these genes were predominantly associated with the MAPK signalling pathway, NF‐kappaB signalling pathway, and inflammatory signalling pathway (Figure 1J).

### 3.2 GSEA Analysis and Immune Infiltration Analysis

**FIGURE 2:**
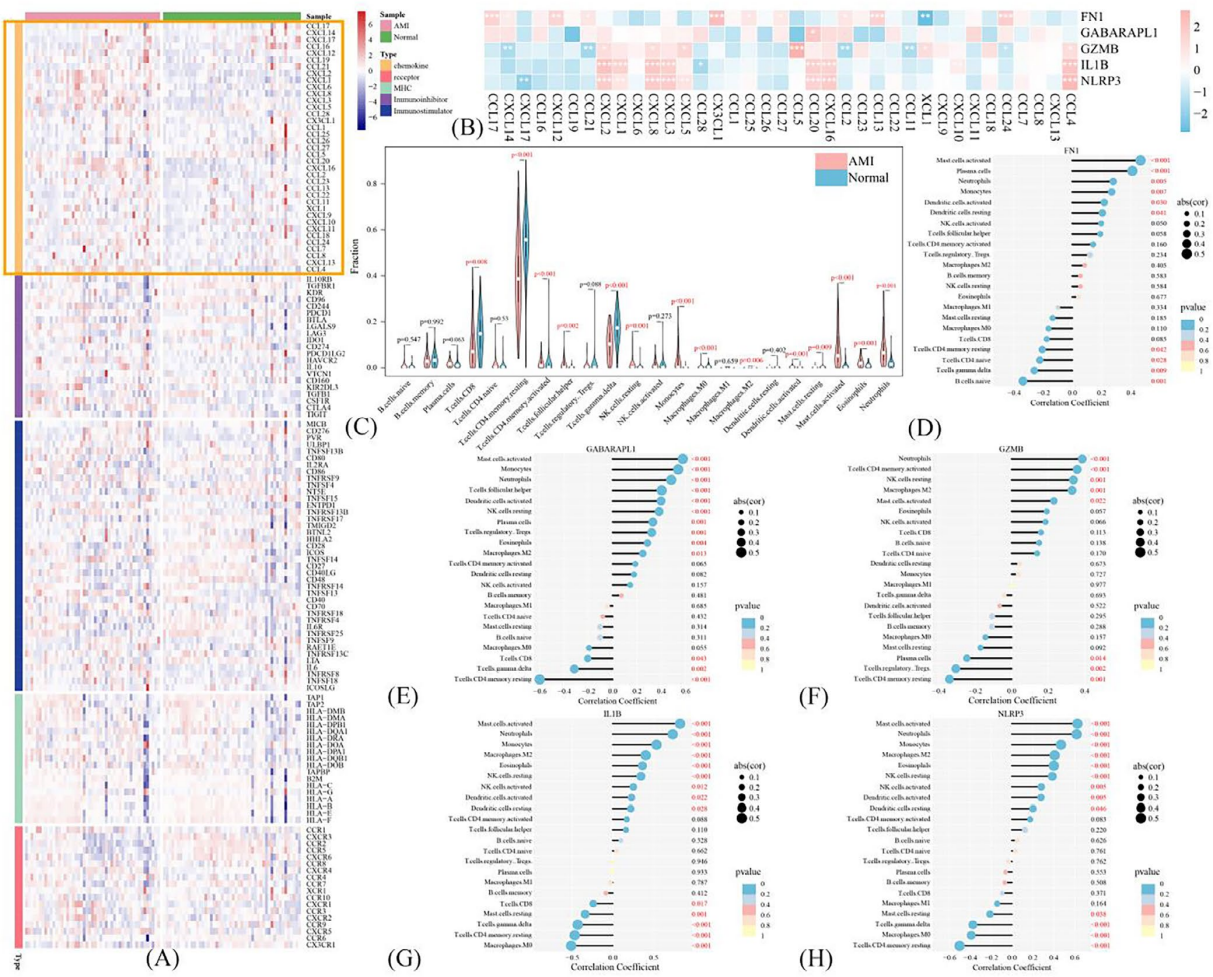
GSEA analysis and Immune infiltration analysis. (A) The heatmap of chemokine, Immunostimulator, Immunoinhibitor, MHC and receptor in the GSE66360 dataset. (B) Correlation analysis of FN1, GABARAPL1, GZMB, NLRP3 and IL1β with chemokine in the GSE66360 dataset. (C) The Violin plot of the immune cells in the GSE66360 dataset. (D–H) Correlation analysis of FN1, GABARAPL1, GZMB, NLRP3 and IL1β with immune cells in the GSE66360 dataset.

To further explore the function of the five genes, the GSEA analysis was employed to identify biological processes and signalling pathways associated with them in MI patients. These five genes were found to be significantly enriched in autophagy, pyroptosis, and inflammatory processes (Figure S2A–J). The aforementioned findings collectively confirmed the close association between these five genes and the inflammatory response. Consequently, we proceeded to conduct an immune infiltration analysis. We first detected the expression levels of chemokine, immunostimulator, immunoinhibitor, MHC, and receptor‐related genes in both MI and Control samples. Our findings revealed that immune‐related genes within the chemokine group were expressed at significantly higher levels in MI samples compared with those in Control samples (Figure 2A). By analysing the correlation between the five genes and chemokine group genes, we observed a positive correlation between IL1β, NLRP3, and GZMB with CCL4, CXCL8, and CXCL2 respectively, while GABARAPL1 was positively correlated with CCL20 (Figure 2B). The violin plots for immune cell content comparison between MI and Control samples showed significant increases in monocyte and neutrophil proportions among MI patients (Figure 2C). Spearman correlation analysis further demonstrated high correlations between these five genes with neutrophils as well as monocytes (Figure 2D–H).

### 3.3 ROC Curve and Nomogram Analysis

**FIGURE 3:**
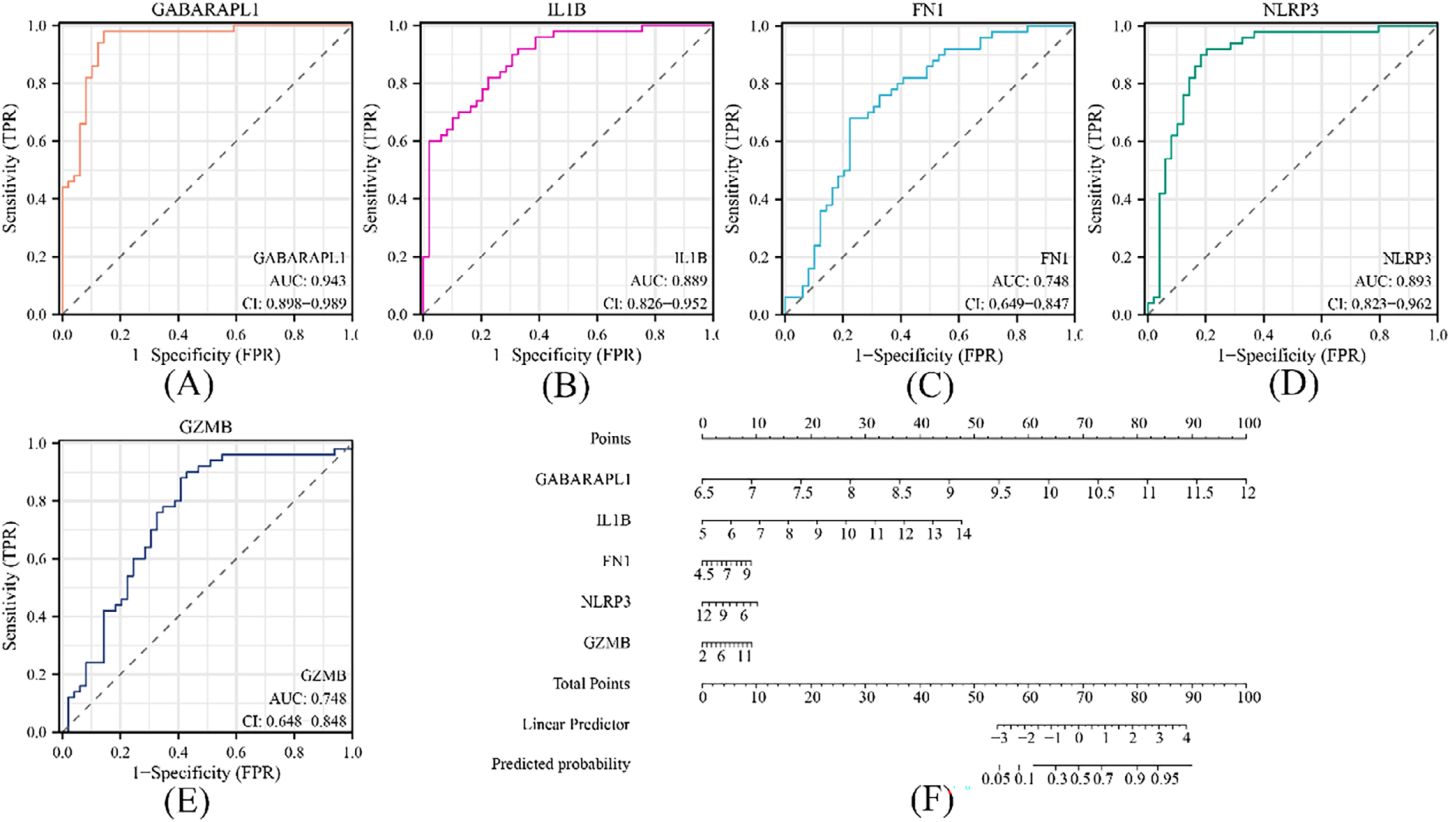
ROC curve and Nomogram analysis. (A‐E) The ROC curve for evaluating the diagnostic efficacy of GABARAPL1, IL1β, FN1, NLRP3, and GZMB. (F) Construction of a nomogram for predicting the risk of the five pivotal genes based on the SVM model.

The “pROC” package was utilised to generate a ROC curve to further validate the diagnostic value of these top five important genes. Among them, GABARAPL1 exhibited the highest accuracy (AUC = 0.943), followed by IL1β (AUC = 0.889), NLRP3 (AUC = 0.893), FN1 (AUC = 0.748) and GZMB (AUC = 0.748) (Figure 3A–E). Additionally, a nomogram incorporating these five significant risk factors was constructed for predicting MI, highlighting the diagnostic value and intervention target of GABARAPL1 in predicting its occurrence (Figure 3F).

### 3.4 GABARAPL1 Expression Is Increased in the Myocardium Post‐MI Mice

**FIGURE 4:**
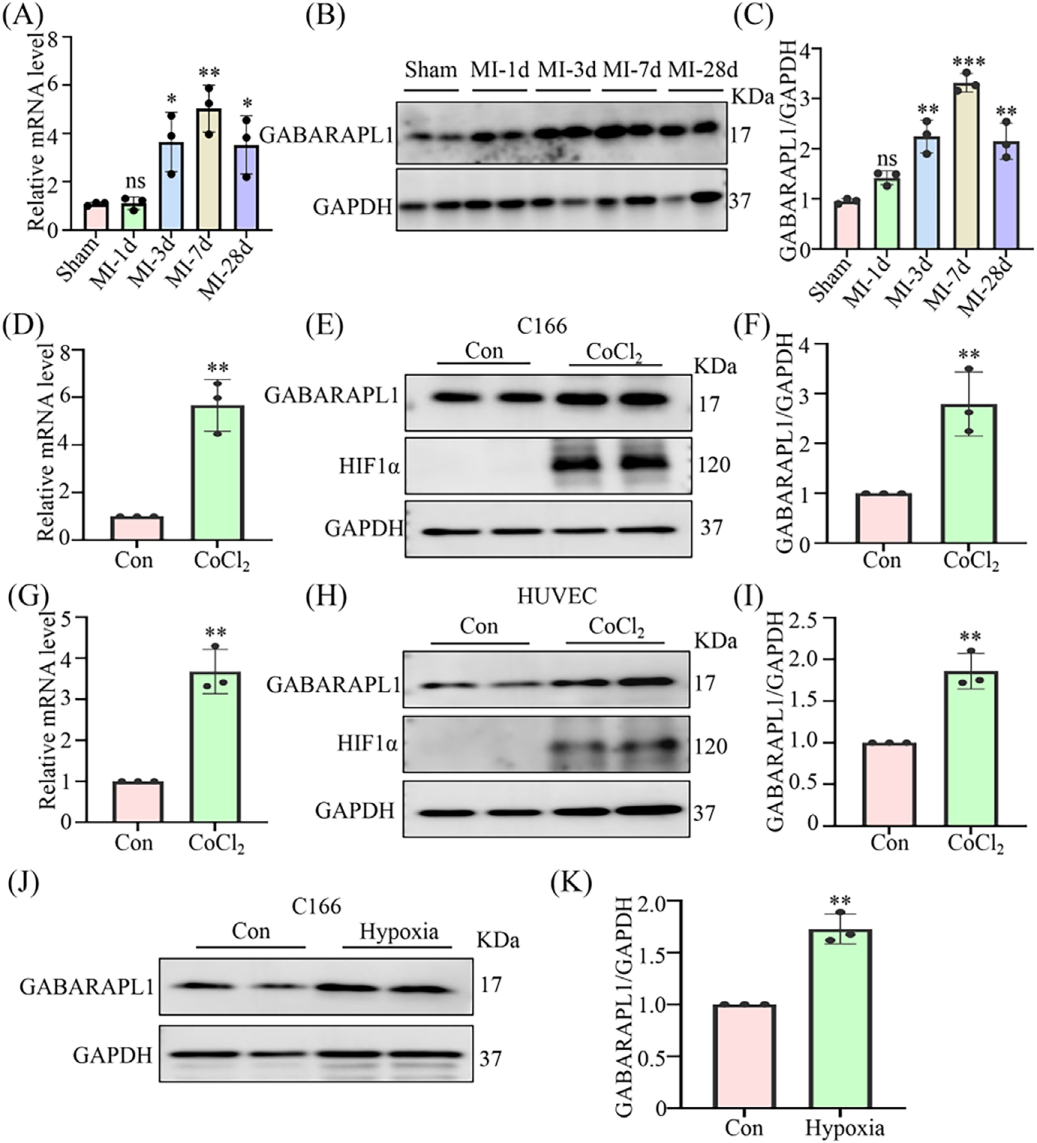
GABARAPL1 expression is increased in the myocardium of post‐MI mice. (A–C) qRT‐PCR and Western blotting analyses of *GABARAPL1* expression in mouse heart tissues at the indicated time points post‐MI. *p < 0.05, **p < 0.01, ***p < 0.001 vs. Sham group; *n* = 3. (D–F) qRT‐PCR and Western blotting analyses of *GABARAPL1* expression in C166 cells with or without CoCl_2_. **p < 0.01 vs. Con group; *n* = 3. (G–I) qRT‐PCR and Western blotting analyses of GABARAPL1 expression in HUVEC cells with or without CoCl_2_; HIF1a was used as a marker of hypoxia. **p < 0.01 vs. Con group; *n* = 3. (J, K) Western blotting analyses of GABARAPL1 expression in C166 cells with or without hypoxia. **p < 0.01 vs. Con group; *n* = 3. Data were presented as mean ± SD. Statistical significance was assessed by one‐way ANOVA and Student's *t*‐test.

We further validated the expression of GABARAPL1 in MI mice. qRT‐PCR results demonstrated that the mRNA level of *GABARAPL1* was increased on the 1st day post‐MI and peaked on the 7th day (Figure 4A). Western blotting analysis at various time points after MI yielded similar outcomes (Figure 4B,C). Moreover, the expression levels of GABARAPL1 across various cell lines were evaluated using Western blotting, which demonstrated that endothelial cells exhibited significantly higher expression of GABARAPL1 compared with other cell lines under physiological conditions (Figure S3A). We subsequently investigated the expression levels of GABARAPL1 in endothelial cells under hypoxia. Initially, C166 cells were stimulated with varying concentrations of CoCl_2_ (0, 400, 600, 800 μM) for 24 h. The findings revealed that GABARAPL1 expression reached its peak at a concentration of 600 μM, thus establishing it as the optimal dosage (Figure S4A). Subsequently, C166 cells were exposed to 600 μM CoCl_2_ to simulate an anoxic environment, which resulted in a significant increase of GABARAPL1 in both protein and mRNA levels (Figure 4D–F). Furthermore, we confirmed these observations using HUVEC cells and obtained consistent results (Figure 4G–I). Simultaneously, we observed a significant upregulation of GABARAPL1 expression in endothelial cells under the condition of physical hypoxia (Figure 4J,K). In view of its elevated expression in MI‐induced cardiac tissue and hypoxia‐induced endothelial cells, we proposed that GABARAPL1 may play a pivotal role in the pathogenesis of MI.

### 3.5 GABARAPL1 Modulates Hypoxia‐Induced Pyroptosis in Endothelial Cells

**FIGURE 5:**
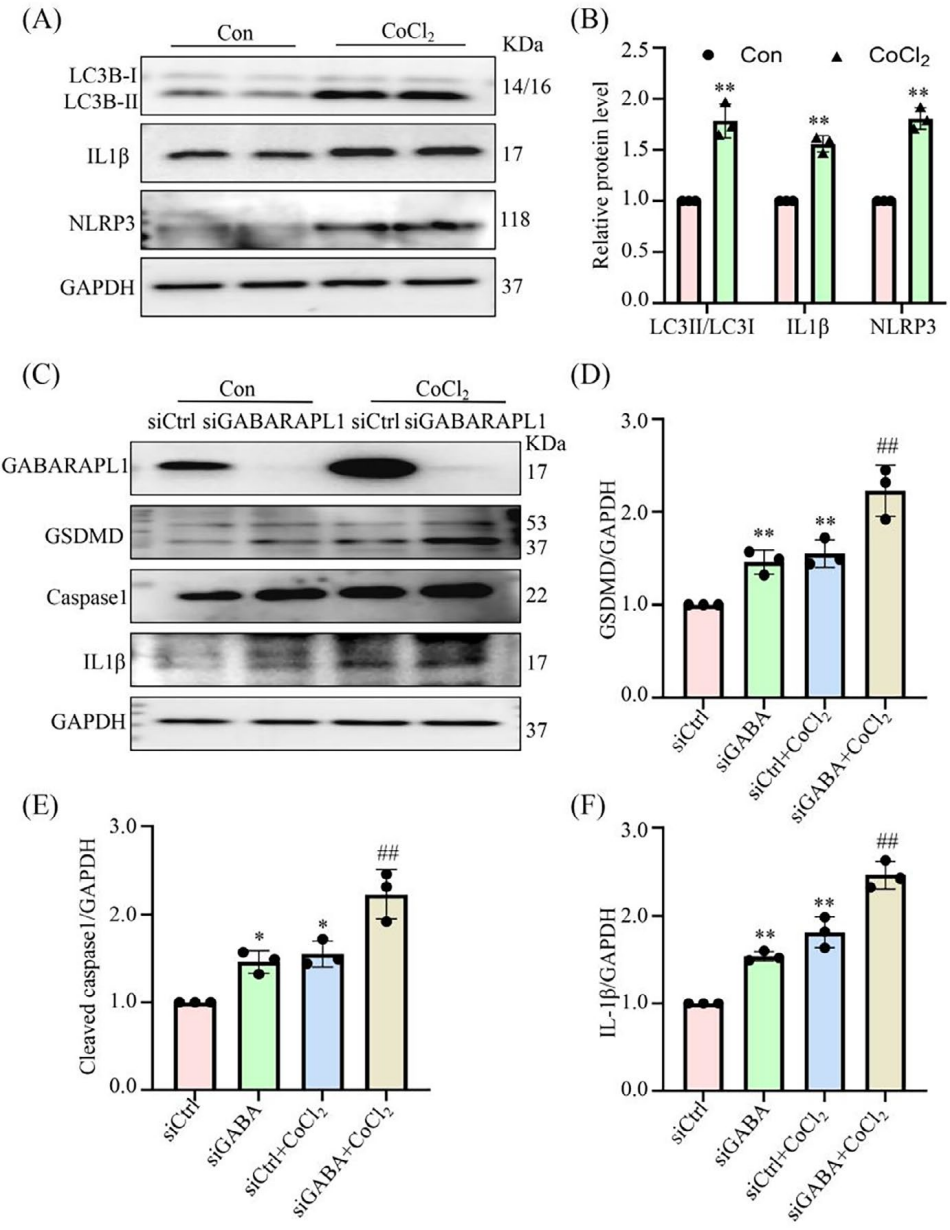
GABARAPL1 modulates hypoxic‐induced pyroptosis in endothelial cells. (A,B) Western blotting analyses of LC3B, NLRP3, and IL1β expression in endothelial cells with or without CoCl_2_. **p < 0.01 vs. Con group; *n* = 3. (C–F) Western blotting analyses showing that the expression of pyroptosis factors was significantly increased under hypoxia, which was markedly aggravated by siGABARAPL1. **p < 0.01, ***p < 0.001 vs. siCtrl group; ^##^ *p* < 0.01 vs. siCtrl + CoCl_2_ group; *n* = 3. Data were presented as mean ± SD. Statistical significance was assessed by one‐way ANOVA and Student's t‐test.

The involvement of pyroptosis is widely implicated in the crucial process of MI. Consistent with previous research, Western blotting data revealed a significant upregulation in the expression levels of pyroptosis markers, including IL1β and NLRP3 in hypoxia‐induced endothelial cells, along with the autophagy marker gene LC3I/II (Figure 5A,B). We subsequently investigated the role of GABARAPL1 in hypoxia‐induced pyroptosis of endothelial cells. Our findings revealed a significant increase in hypoxia‐induced pyroptosis, and the knockdown of GABARAPL1 further exacerbated hypoxia‐induced pyroptosis. Collectively, these results underscored the pivotal function of GABARAPL1 in modulating the pyroptotic process following MI (Figure 5C–F).

### 3.6 Prediction of Potential Drug and Analysis of Molecular Docking

We further employed CMap to identify potential drug candidates for GABARAPL1. In particular, the CMap scores exhibited a negative correlation with the therapeutic efficacy of the drugs. Subsequently, we screened and analysed 20 drugs that were most closely associated with GABARAPL1, utilising a heat map to elucidate their underlying mechanisms of action (MOA) in MI patients (Figure S5A). The Radar Chart illustrates the CMap scores of the selected 20 drugs (Figure S15B). Autodock Vina software was utilised to investigate the binding affinity between these potential molecular compounds and GABARAPL1. Among them, PIK90 exhibited satisfactory binding activity with GABARAPL1 with a binding energy of −6.4 Kcal/mol (Table S3). Ultimately, PIK90 was identified as the most potential target drug for GABARAPL1. Molecular docking analysis further revealed that PIK90 interacted with GABARAPL1 by forming conventional hydrogen bonds with ARG47 and GLU24, and alkyl bonds with LYS48, and ALA26 (Figure S5C).

### 3.7 PIK90 Treatment Significantly Alleviates Cardiac Dysfunction and Myocardial Fibrosis Induced by MI

**FIGURE 6:**
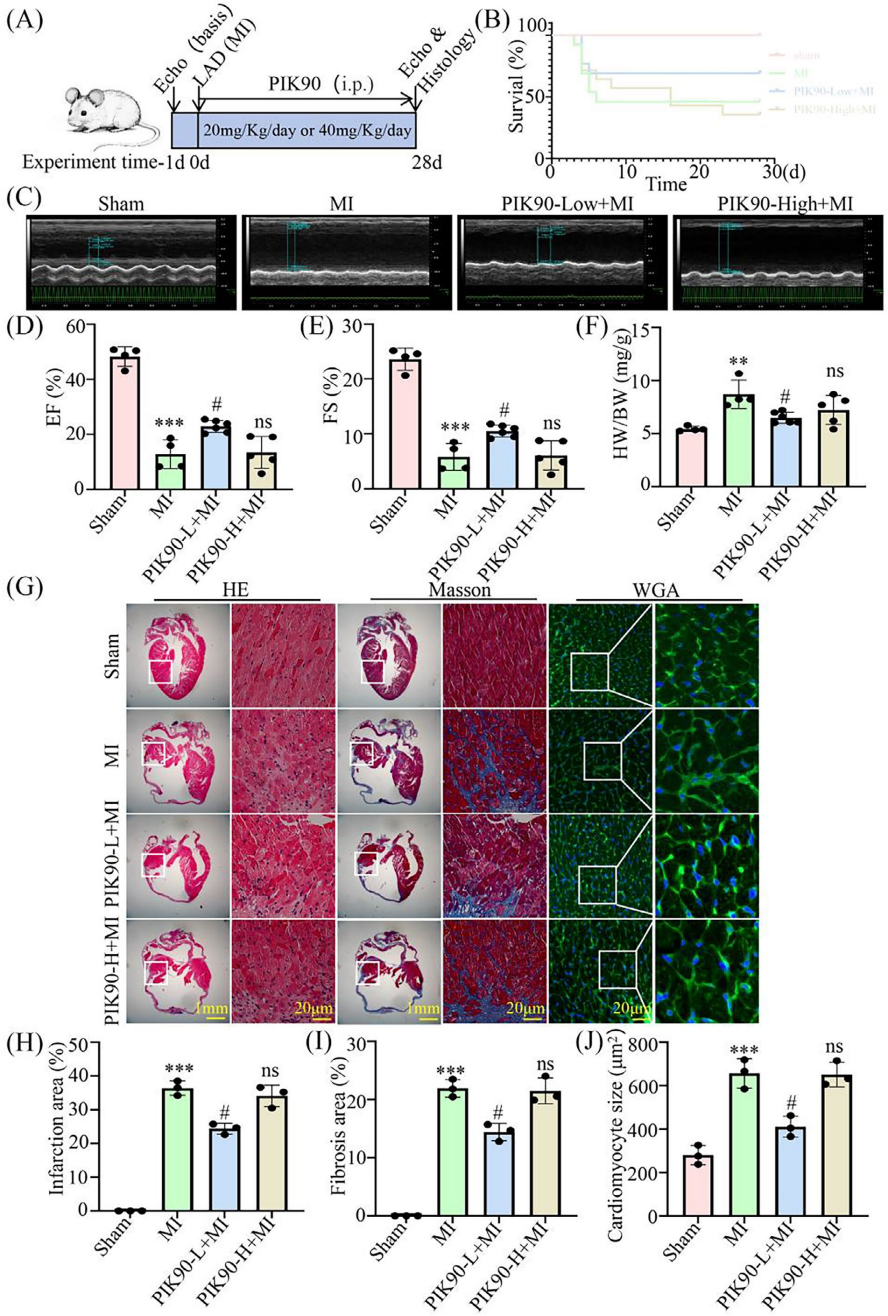
PIK90 treatment significantly alleviates cardiac dysfunction and myocardial fibrosis induced by MI. (A) The timeline of the experimental design for PIK90 treatment. (B) Survival rate analysis. (C) Representative echocardiographic images on the 28 days post‐MI. (D) Echocardiographic analysis of Ejection Fraction (EF). ***p < 0.001 vs. Sham group; ^#^ *p* < 0.05 vs. MI group; ns (no significance) vs. MI group; *n* = 4–6. (E) Echocardiographic analysis of Fractional Shortening (FS). ***p < 0.001 vs. Sham group; ^#^ *p* < 0.05 vs. MI group; ns (no significance) vs. MI group; *n* = 4–6. (F) The analysis of heart weight (HW) and body weight (BW) ratio. **p < 0.01 vs. Sham group; ^#^ *p* < 0.05 vs. MI group; ns (no significance) vs. MI group; *n* = 4–6. (G) The Hematoxylin and Eosin (H&E) staining, Masson staining, and Wheat Germ Agglutinin (WGA) staining of adult mouse hearts at 28 days post MI, scale bar = 1 mm or 20 μm. (H–J) Quantitative analysis of infarction area (H), fibrosis area (I) and Cardiomyocyte size (J). ***p < 0.001 vs. Sham group; ^##^ *p* < 0.01 vs. MI group; ns (no significance) vs. MI group; *n* = 3. Data were presented as mean ± SD. Statistical significance was assessed by one‐way ANOVA.

To evaluate the efficacy of PIK90 on cardiac function following MI, the C57BL/6J mice following MI were treated with PIK90 (20 mg/kg/day or 40 mg/kg/day) via intraperitoneal injection (Figure 6A). Survival analysis demonstrated a significant enhancement in survival rates in the PIK90‐Low + MI group compared with the MI group, whereas no substantial changes were observed in the PIK90‐High + MI group (Figure 6B). Additionally, echocardiographic assessments revealed that treatment with 20 mg/kg/day of PIK90 led to significant improvements in cardiac function, while no such improvements were noted in the high‐dose group (Figure 6C–F). The extent of myocardial injury was evaluated using H&E, Masson, and WGA staining. The results indicated that, compared to the MI group, the infarct size, myocardial fibrosis, and cross‐sectional area of cardiomyocytes in the infarct margin zone were reduced in the PIK90‐Low + MI group, while no differences were observed in the PIK90‐High + MI group (Figure 6G–J). Collectively, these findings suggest that low doses of PIK90 can effectively improve cardiac dysfunction and mitigate myocardial fibrosis following MI.

### 3.8 PIK90 Upregulates GABARAPL1 Expression and Mitigates Myocardial Pyroptosis Induced by MI

**FIGURE 7:**
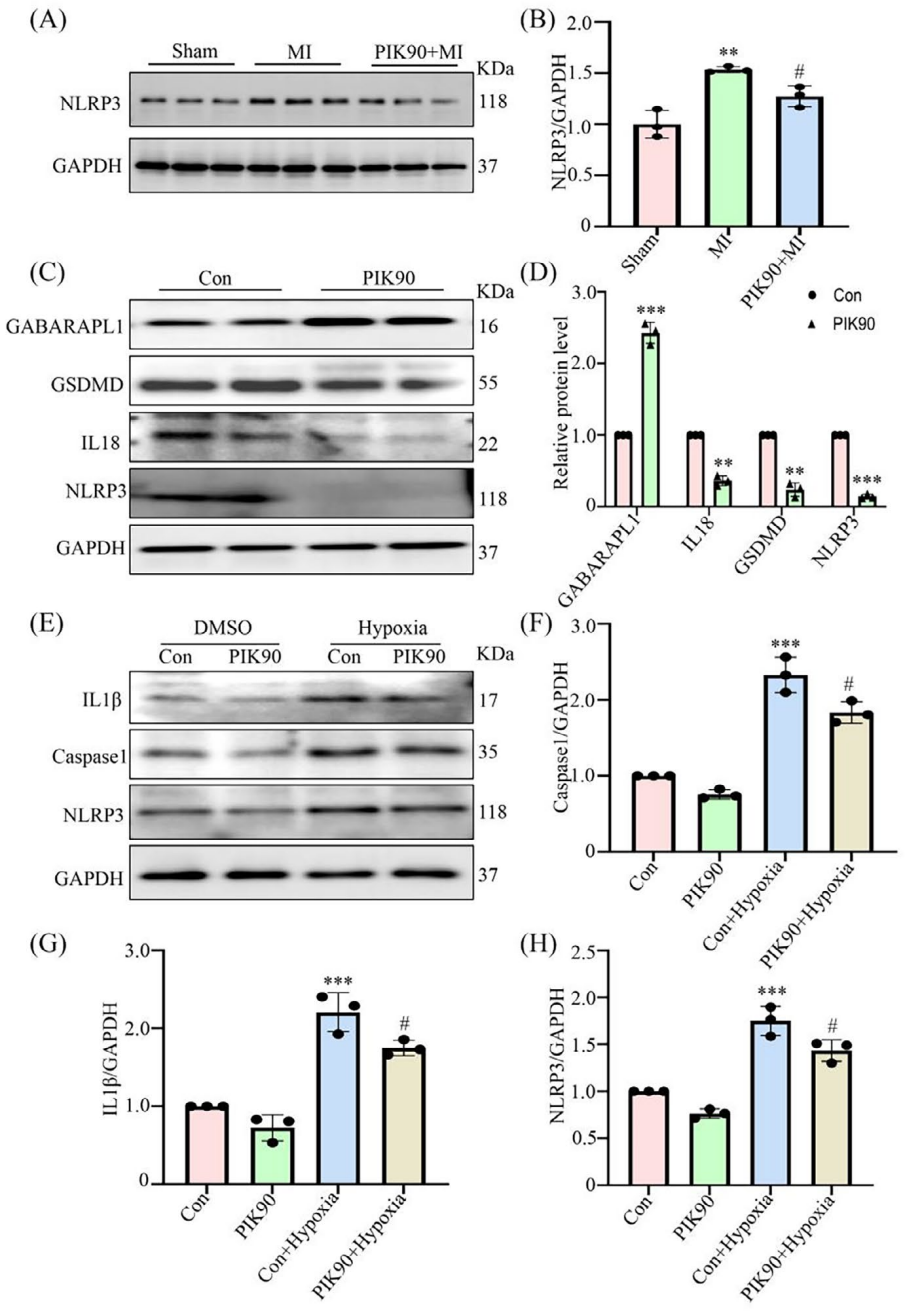
PIK90 upregulates GABARAPL1 expression and mitigates myocardial pyroptosis induced by MI. (A, B) Western blotting analysis showing that the expression of NLRP3 under the treatment of PIK90. **p < 0.01 vs. Sham group; ^#^ *p* < 0.05 vs. MI group; *n* = 3. (C, D) Western blotting analysis showing that PIK90 inhibited the expression of GSDMD, IL18, and NLRP3. **p < 0.01, ***p < 0.001 vs. Con group; *n* = 3. (E–H) Western blotting analysis showing that the expression of IL1β, Caspase1, and NLRP3 under hypoxia was significantly decreased with the treatment of PIK90. ***p < 0.001 vs. Con group; ^#^ *p* < 0.05 vs. Con+Hypoxia group; *n* = 3. Data were presented as mean ± SD. Statistical significance was assessed by one‐way ANOVA and Student's *t*‐test.

We subsequently investigated the effects of PIK90 on GABARAPL1 and its regulatory role in MI‐induced pyroptosis. In vivo, Western blotting results demonstrated a significant increase in the expression levels of NLRP3 in the MI group compared with the Sham group, while treatment with PIK90 markedly attenuated MI‐induced pyroptosis (Figure 7A,B). In vitro, the results demonstrated that PIK90 upregulated the expression of GABARAPL1 while downregulating the expression of GSDMD, IL18 and NLRP3 in endothelial cells under physiological conditions (Figure 7C,D). Subsequently, the impact of PIK90 on hypoxia‐induced pyroptosis was investigated. The results revealed that hypoxia led to a significant upregulation of NLRP3, Caspase1 and IL1β compared with the Control group, while PIK90 treatment markedly attenuated hypoxia‐induced pyroptosis in endothelial cells (Figure 7E–H). These results indicated that PIK90 increased the expression of GABARAPL1 and modulated hypoxia‐induced pyroptosis in endothelial cells.

## 4 Discussion

The prevalence of cardiovascular disease continues to be the primary cause of global mortality and places a substantial burden on individuals' lives and overall health. MI is a significant contributor to the increased mortality associated with cardiovascular disease, characterised by high morbidity and unfavourable prognosis, thus posing a substantial threat to human well‐being [21]. Although thrombolysis and percutaneous coronary intervention (PCI) have significantly reduced the mortality rates associated with MI, the complications arising from MI still markedly impair patients' quality of life and long‐term prognosis [22, 23]. Therefore, it is imperative to ascertain biomarkers with high sensitivity and specificity to reduce MI incidence and improve patient prognosis. Autophagy and pyroptosis play pivotal roles in the pathogenesis of MI. Nevertheless, our comprehension of the mechanisms underlying pyroptosis and autophagy in MI remains limited. In this study, we employed bioinformatics to extract valuable insights from MI datasets, with the objective of elucidating the expression patterns and associated molecular mechanisms governing pyroptosis and autophagy in MI.

In this study, we first identified five important genes, including GABARAPL1, GZMB, IL1β, NLRP3, and FN1, which are significantly associated with pyroptosis, and autophagy in MI. Further, the GSEA analysis showed that these five genes were not only enriched in the autophagy and pyroptosis pathway, but also enriched, in the inflammation‐related pathway in MI. In light of the substantial association between these genes and inflammatory signalling pathways, we then performed immune infiltration analysis. The results revealed a significant increase in chemokine expression in MI samples, with a strong positive correlation between the five genes and immune‐related chemokine genes, such as CCL4, CCL5, CXCL2, and CCL20. Chemokines are a family of chemotactic cytokines that play a key role in the sequential mobilisation of immune cell subsets and are key drivers of cardiac repair and remodelling after MI [24, 25]. However, abnormal chemokine expression may exacerbate the inflammatory response. It has been reported that the increasing secretion of CCL4 and CCL5 could lead to cardiac function impairment after MI in mice [26]. Additionally, increasing evidence indicated that the expression of CXCL2 and CXCL5 chemokines can be inhibited to suppress the recruitment of neutrophils to damaged myocardium and provide cardiac protection [27]. CCL20 expression is elevated in infarcted tissue and miR‐19a targets CCL20 directly to eliminate MI‐induced cardiomyocyte injury [28]. In addition, the results revealed that these genes exhibited positive correlations with neutrophils and monocytes while demonstrating negative correlations with T cells and CD4^+^ T cells, suggesting their potential role in modulating the recruitment of immune cells, particularly neutrophils and monocytes. Currently, the crucial role of monocytes and neutrophils in cardiovascular disease has been extensively studied. Studies have shown that inhibiting haematopoietic stem cell generation and monocyte recruitment to plaque sites can impede the progression of post‐MI atherosclerosis [29]. It has been reported that inhibiting neutrophil inflammation can improve coronary microvascular perfusion and reduce infarct size in I/R mice [30]. Subsequently, a predictive model was developed, and the results demonstrated that GABARAPL1 exhibited significant diagnostic potential, thereby identifying it as the hub gene.

GABARAPL1 was ultimately identified as the hub gene with the highest diagnostic value, which may play a crucial role in mediating the crosstalk between autophagy and pyroptosis during the development of MI. GABARAPL1, also known as autophagy‐related 8 (ATG8) or Glandular epithelial cell protein 1 (GEC1), exhibits expression in various tissues across mice, rats, and humans. Notably, its expression is particularly prominent in cardiac tissues [31, 32]. The expression of GABARAPL1 is upregulated in response to tumour‐induced hypoxia, thereby enhancing the secretion of exosomes and growth factors, consequently facilitating tumour angiogenesis [33]. This is consistent with our findings that the expression of GABARAPL1 is also upregulated in damaged cardiac tissue and hypoxia‐induced endothelial cells. The inhibition of GABARAPL1 in breast cancer cells has been reported to result in a reduction in autophagy flux and impaired clearance of damaged mitochondria [34]. Pyroptosis is a highly inflammatory form of programmed necrosis that plays a critical role in cardiomyocyte injury induced by IR [35]. In addition, a substantial body of research has confirmed that the activation of autophagy exerts a protective effect on cardiac injury induced by various cardiovascular diseases [36, 37]. It has been demonstrated that the activation of autophagy exerts a protective effect against IR‐induced cardiac injury through the inhibition of inflammatory responses [38]. Thus, we further elucidated the role of GABARAPL1 in regulating MI‐induced pyroptosis through both in vivo and in vitro experiments. The results revealed that a significant upregulation of GABARAPL1 expression in myocardial tissues induced by MI, as well as in hypoxia‐induced endothelial cells. Furthermore, silencing of GABARAPL1 notably exacerbated hypoxia‐induced pyroptosis in endothelial cells. These findings suggest that GABARAPL1 may alleviate MI by attenuating hypoxia‐induced pyroptosis in endothelial cells.

Drug predictions were conducted to identify small molecule compounds targeting the hub gene. As a result, PIK90 was specifically identified as small molecular compounds interacting with GABARAPL1. PIK90 has been shown in previous studies to significantly attenuate CXCL12‐induced chemotaxis and actin polymerisation, while also demonstrating efficacy in inducing apoptosis in chronic lymphocytic leukaemia cells [39]. In our study, we demonstrated that PIK90 plays a crucial role in mitigating myocardial injury induced by MI. Additionally, we found that it can mitigate hypoxia‐induced pyroptosis in endothelial cells. In conclusion, PIK90 demonstrated therapeutic value for MI and may therefore emerge as targeted drugs for future heart attack treatments.

Our current study is based on bioinformatics and has been validated both in vivo and in vitro. However, further clinical evidence is still required to confirm its utility as a predictor of MI, as well as a target for prognosis and treatment for MI. Furthermore, additional experiments are necessary to elucidate the specific regulatory mechanisms of *GABARAPL1* involved in the pathogenesis of autophagy and pyroptosis in MI.

## 5 Conclusion

In this study, GABARAPL1 has been identified as a critical biomarker and potential therapeutic target for MI. We have developed and validated a predictive model both in vivo and in vitro. In addition, our findings indicate that PIK90 can mitigate myocardial injury and endothelial cell pyroptosis induced by MI, suggesting its potential as a therapeutic agent for the condition. Consequently, this study offers significant insights into the mechanisms of autophagy and pyroptosis in MI and highlights a promising avenue for future clinical intervention.

## Author Contributions

**Chunying Liu:** writing – original draft (equal). **Chenghui Yan:** validation (equal). **Dan Liu:** methodology (equal). **Haixu Song:** writing – review and editing (equal). **Yaling Han:** project administration (equal), writing – review and editing (equal).

## Conflicts of Interest

The authors declare no conflicts of interest.

## Supporting information


Data S1.

## Data Availability

The GSE66360 dataset was obtained from the GEO database (https://www.ncbi.nlm.nih.gov/gds/). The pyroptosis‐related genes were obtained from the MSIGDB database (http://www.gsea‐msigdb.org/gsea/msigdb/) and the autophagy‐related genes were obtained from the HAMdb2 database (http://www.autophagy.lu/index.html).
