# Supplementary material for: GABARAPL1 Exerts Regulatory Effects on Hypoxia‐Induced Pyroptosis in the Pathogenesis of Myocardial Infarction
Source: J Cell Mol Med. 2025 Mar 17;29(6):e70469. doi: 10.1111/jcmm.70469 (PMC11913011; doi:10.1111/jcmm.70469)
Supplement: Supplementary file 1 — Data S1. [file JCMM-29-e70469-s001.docx]

Supplementary Material

***GABARAPL1* exerts regulatory effects on hypoxia-induced pyroptosis in the pathogenesis of myocardial infarction**

**Figure S1.**

**
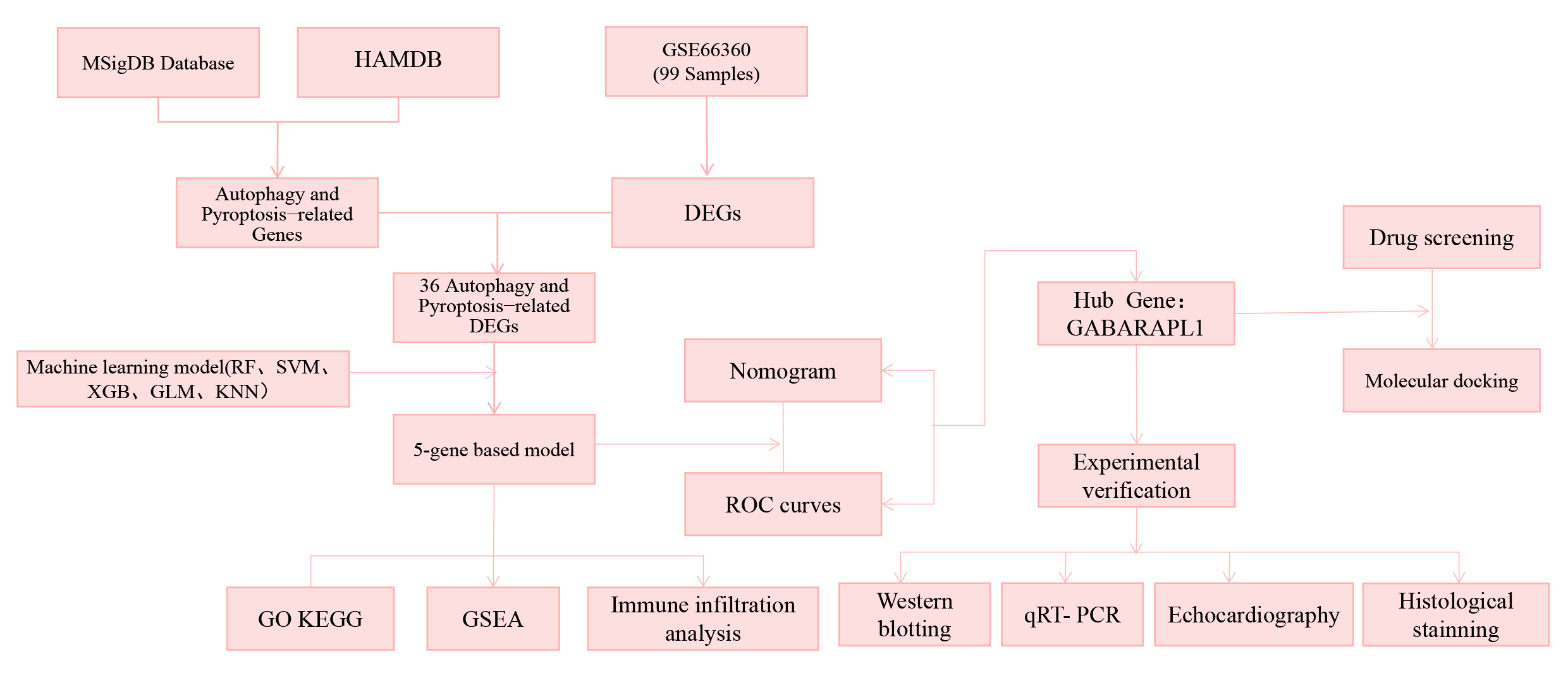
**

**Figure S1. Inclusion of Literature Flowchart**

**Figure S2.**

**
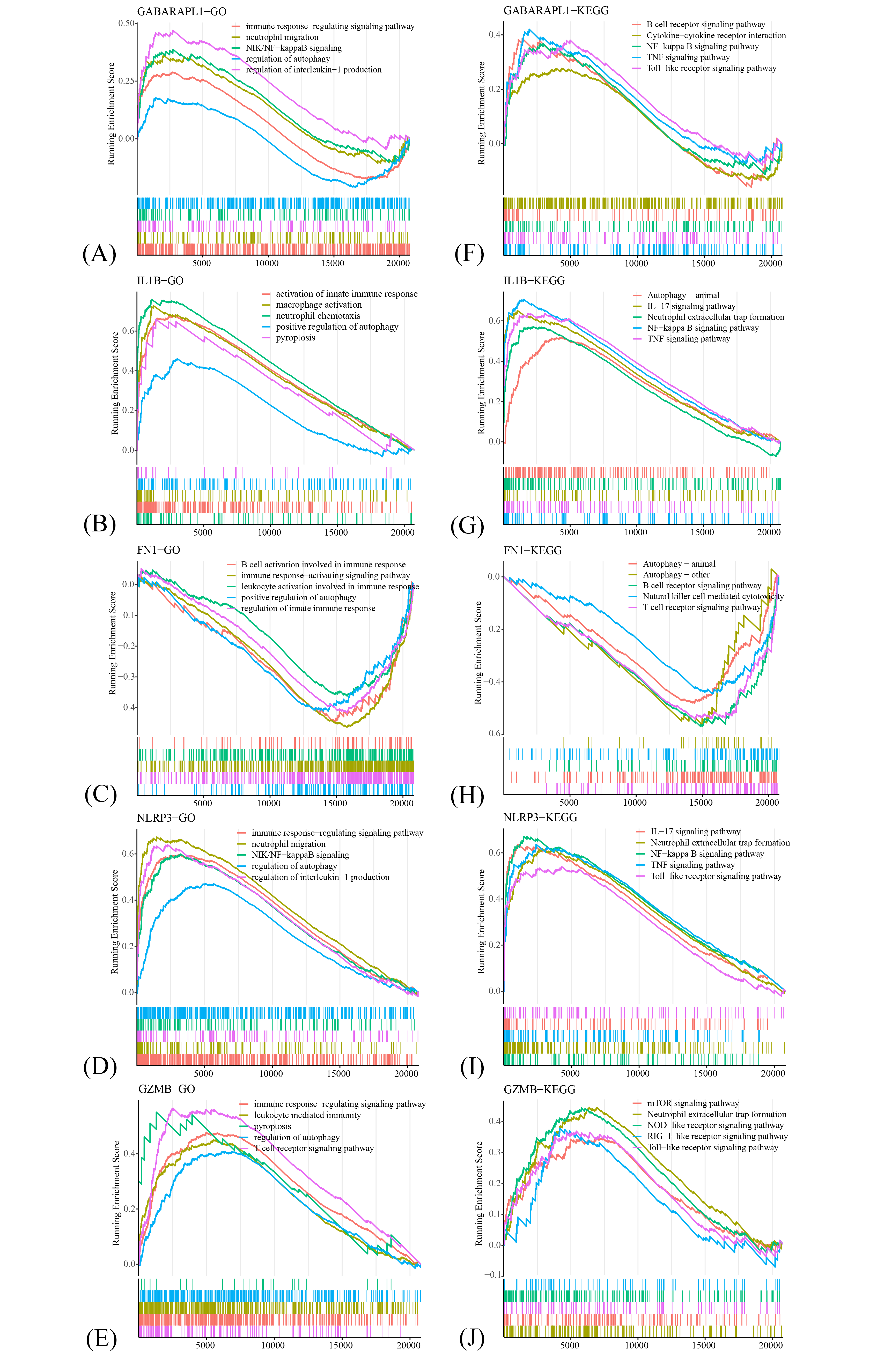
**

**Figure S2**. **Gene Set Enrichment Analysis (GSEA) of pivotal Genes. (A–E)** GABARAPL1, GZMB, IL1β, NLRP3 and FN1 enrichment analyses of BP. **(F-J)** GABARAPL1, GZMB, IL1β, NLRP3 and FN1 enrichment analyses of KEGG.

**Figure S3.**


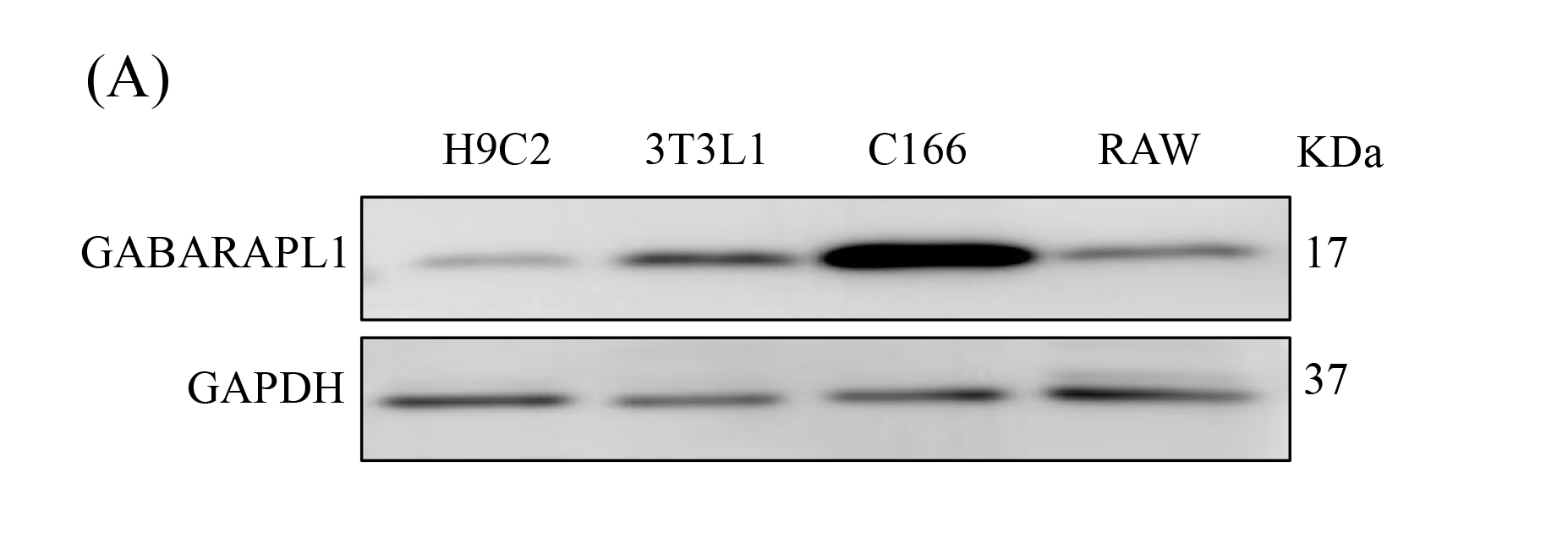


**Figure S3. The cellular expression profile of *GABARAPL1*. (A)** The physiological expression of *GABARAPL1* in various cell lines.

**Figure S4.**


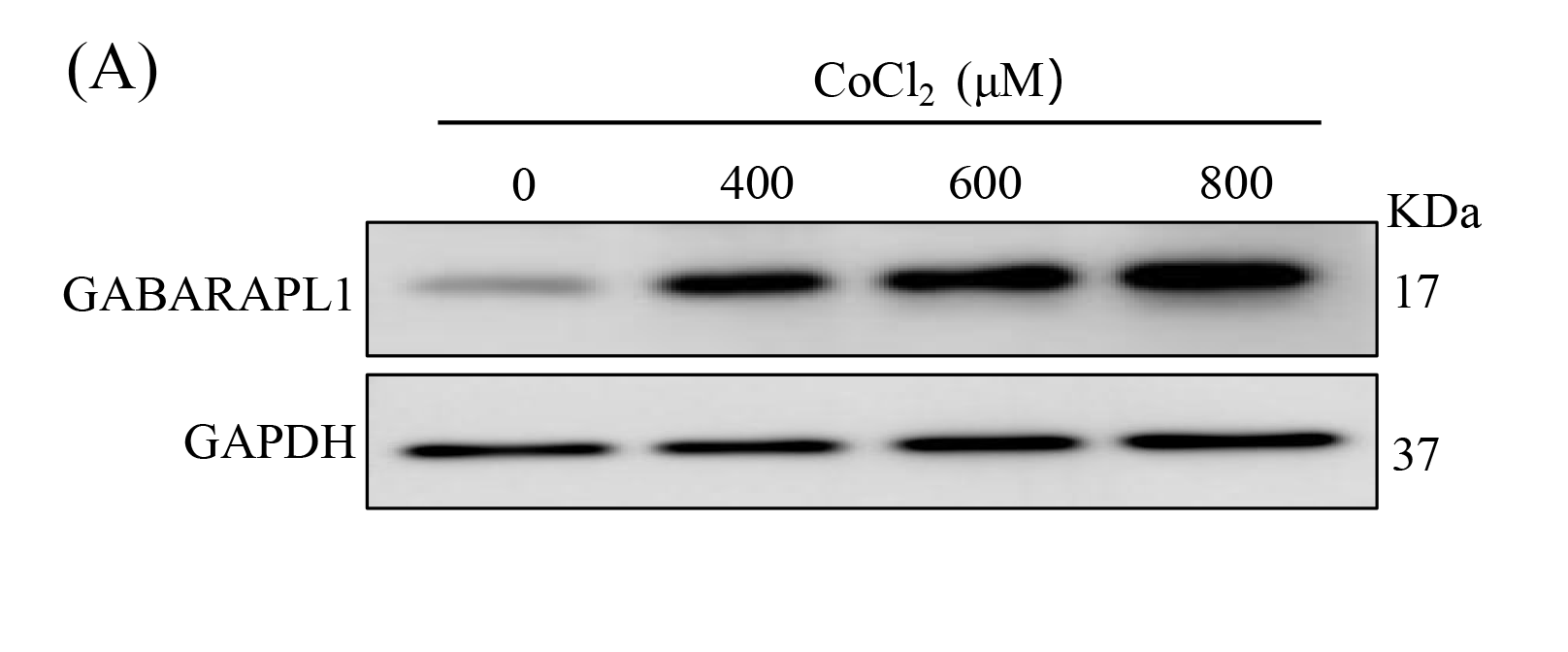


**Figure S4. Screening for the Optimal Concentration of CoCl_2_.** **(A)** The expression of *GABARAPL1* was assessed in response to stimulation with 0, 400, 600, and 800 µM CoCl2.

**Figure S5.**

**
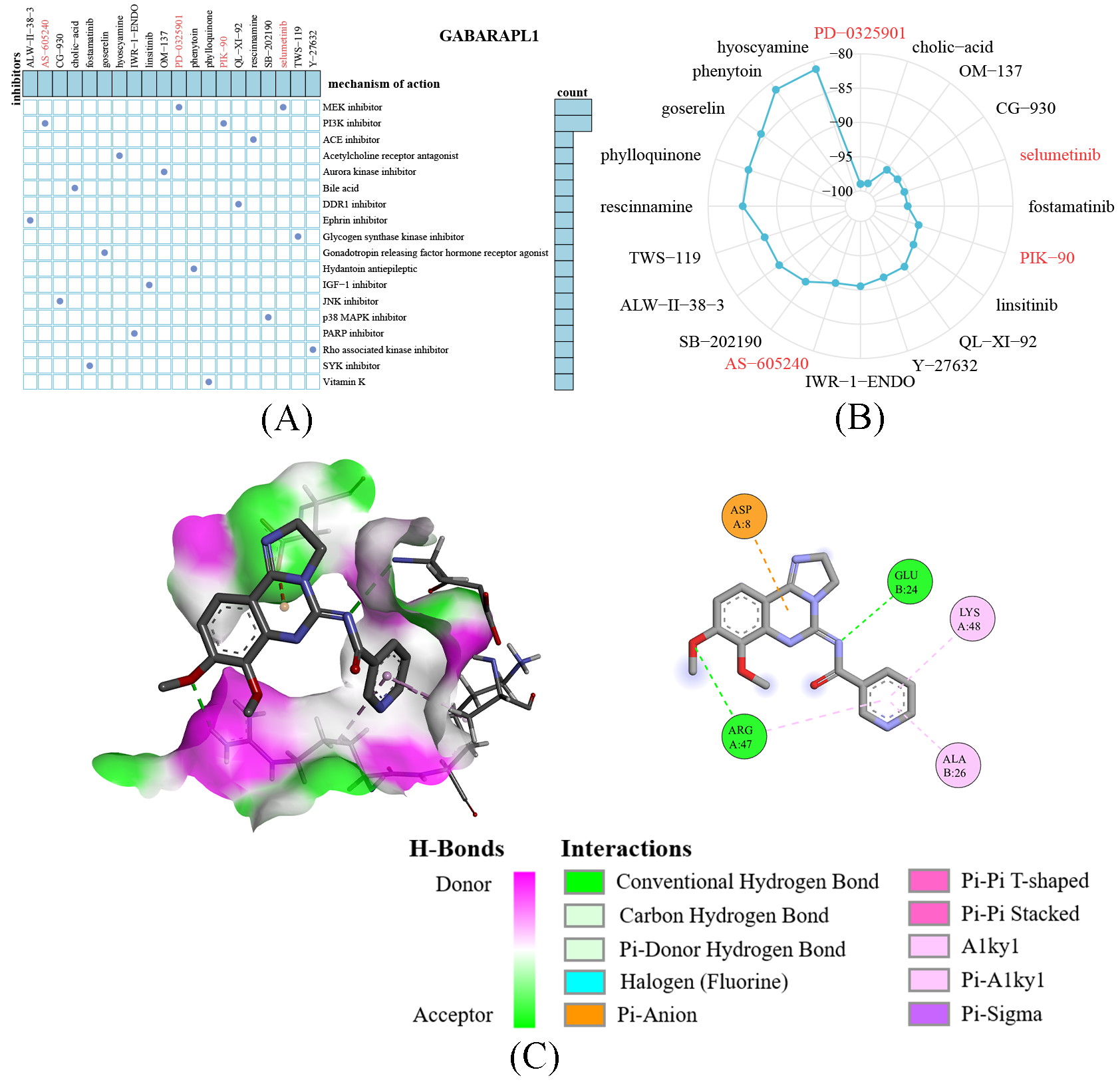
**

**Figure S5.** **Prediction of potential Drug and analysis of molecular docking. (A)** Heatmap showed candidate drugs and relevant mechanisms of GABARAPL1. **(B)** The Radar Chart of 20 targeted drugs. **(C)** Molecular docking pattern between PIK90 and GABARAPL1*.*

**Table S1. siRNA sequences of primers are as follows**

| Target | sense sequence (5′ → 3′) | antisense sequence (3′ → 5′) |
| --- | --- | --- |
| GABARAPL1 | GAUGGGAGCACCUGGACUUTT | AAGUCCAGGUGCUCCCAUCTT |
| Control | UUCUCCGAACGUGUCACGUTT | ACGUGACACGUUCGGAGAATT |

**Table S2. Primer Sequence**

| Primer | Sequence (5′ → 3′) | |
| --- | --- | --- |
| *GABARAPL1*(Mouse) | Forward | CCTCTGTCTCCATACCTTCCTCTC |
|  | Reverse | CGAATGTCTCCTGCCACAACTG |
| *GABARAPL1*(Human) | Forward | CAGGGTCCCCGTGATTGTAG |
|  | Reverse | GCTAGAGGATGCCAGACTGC |
| *GAPDH* | Forward | GCATCTTCTTGTGCAGTGCC |
|  | Reverse | GATGGTGATGGGTTTCCCGT |

**Table S3. The physicochemical and pharmacokinetic properties typical for four small-molecule drugs**

| Drugs | Binding energy (Kcal/mol) | LogP | HBD | HBA | Mw | PSA | Rotatable bonds |
| --- | --- | --- | --- | --- | --- | --- | --- |
| PIK-90 | -6.4 | 1.3 | 1 | 5 | 351.4 | 88.4 Å2 | 2 |
| PD-0325901 | -6 | 2.64 | 4 | 5 | 482.1 | 90.82Å2 | 7 |
| selumetinib | -5.9 | 3.24 | 3 | 5 | 457.6 | 88.41Å2 | 6 |
| AS-605240 | -5.9 | 2.35 | 1 | 7 | 384.4 | 100.53Å2 | 5 |

Note: LogP, lipid-water partition coefficient; HBD, hydrogen bond donor; HBA, hydrogen bond acceptor; MW, molecular weight; PSA: Polar Surface Area.
